# Disease-Modifying Potential of Metformin and Alendronate in an Experimental Mouse Model of Osteoarthritis

**DOI:** 10.3390/biomedicines9081017

**Published:** 2021-08-15

**Authors:** Lyudmila Belenska-Todorova, Sevdalina Nikolova Lambova, Stela Stoyanova, Elenka Georgieva, Tsvetelina Batsalova, Dzhemal Moten, Desislava Kolchakova, Balik Dzhambazov

**Affiliations:** 1 Faculty of Medicine, Sofia University “St. Kliment Ohridski”, 1407 Sofia, Bulgaria; lbelenska@uni-sofia.bg; 2 Department of Propaedeutics of Internal Diseases, Faculty of Medicine, Medical University of Plovdiv, 4002 Plovdiv, Bulgaria; sevdalina_n@abv.bg; 3 Department in Rheumatology, MHAT “Sveti Mina”, 4000 Plovdiv, Bulgaria; 4 Department of Developmental Biology, Plovdiv University “Paisii Hilendarski”, 4000 Plovdiv, Bulgaria; stela.stoyanova@uni-plovdiv.bg (S.S.); elenkageorgieva@uni-plovdiv.bg (E.G.); tsvetelina@uni-plovdiv.bg (T.B.); moten@uni-plovdiv.bg (D.M.); kolchakova@uni-plovdiv.bg (D.K.)

**Keywords:** osteoarthritis, mouse model, metformin, bisphosphonate, treatment, osteoclastogenesis, adipokines, histopathology

## Abstract

Osteoarthritis (OA) is the most common degenerative joint disease causing progressive damages of the cartilage and subchondral bone, synovial inflammation, and severe pain. Despite the complex pathomorphological changes that occur in OA, the approach to different forms of OA is standardized. The global results from pharmacological treatment are not satisfactory. Hence, this study aimed to explore the effects of metformin, alendronate, and their combination on OA development and progression in mice with collagenase-induced osteoarthritis (CIOA). Female ICR (CD-2) mice were randomized to five groups: control group, CIOA untreated, CIOA + metformin, CIOA + alendronate, and CIOA + metformin + alendronate. OA was induced by the intra-articular (i.a.) injection of collagenase. OA phenotype was analyzed by flow cytometry (bone marrow cell differentiation), ELISA (serum levels of the adipokines leptin and resistin), and histology (pathological changes of the knee joint). Treatment with metformin, alendronate, or their combination inhibited the expression of RANK and RANKL on osteoblasts and osteoclasts obtained by ex vivo cultivation of bone marrow cells in mineralization or osteoclastogenic media. In addition, metformin treatment was effective for the attenuation of fibroblast differentiation, but not of mesenchymal stem cells (MSCs), while alendronate had an opposite effect. The combination of metformin and alendronate had a suppressive effect on both MSCs and fibroblasts differentiation. Treatment with metformin, alendronate, and their combination decreased serum concentrations of leptin and resistin in the chronic phase of arthritis. The histopathological examination showed that compared with the untreated CIOA group (OA score 9), the groups treated with metformin (OA score 4) or alendronate (OA score 6) had lower scores for cartilage changes. Metformin combined with alendronate significantly decreased the degree of cartilage degeneration (OA score 2), suggesting that this combination might be a useful approach for the treatment of OA patients.

## 1. Introduction

Osteoarthritis (OA) is the most common chronic inflammatory degenerative joint disease, leading to disability and negative impacts on physical health and mental wellbeing [1]. It has variable phenotypes in patients, affecting asymmetrically both large and small joints, with the pathological process involving the progressive destruction of the articular cartilage, synovial inflammation, and damage to the subchondral bone, osteophyte formation, joint capsule hypertrophy, development of bone marrow lesions, and changes in ligaments and muscles around the joint [2]. Concepts regarding the disease pathophysiology are evolving, with OA no longer being considered the result of cartilage and bone wear, but rather caused by a multifactorial disorder in which metabolic factors and mild chronic inflammation play central roles [3,4]. 

Current treatment for OA focuses on the symptoms of the disease, but there are still no approved drugs to prevent or slow its progression [5]. The difficulty in establishing a successful disease-modifying treatment is probably related to high disease heterogeneity, i.e., different localizations of the pathological process and different disease phenotypes, as well as complex pathogenesis. Thus, a personalized approach that includes drug combinations which target different pathogenic aspects may be the future key to successful disease-modifying treatment.

Bisphosphonates inhibit bone resorption and therefore they are a major option for the treatment of osteoporosis, however there is not enough evidence for the recommendation of their use in OA. Due to developing knowledge about OA pathogenesis and more specifically about the early changes that occur in the subchondral bone, it has been suggested that it is worth studying the role of antiresorptive bone agents including alendronate as a therapeutic option in OA [6]. Bisphosphonates inhibit osteoclast function and survival, and bone resorption, respectively [7]. 

Alendronate and metformin are substances that appear to be prospective candidates for the development of an effective therapeutic approach for OA. Alendronate possesses the ability to keep subchondral and periarticular bone integral in multiple animal models [8]. Metformin (1,1-dimethylbiguanide) is a member of the biguanide class of compounds, also including phenformin and buformin. Metformin is a first-line therapy for diabetes mellitus type 2 when obesity is present [9]. Metformin’s pharmacologic effect is considered to be due to its ability to activate AMP-activated protein kinase through the reciprocal regulation of Th17 and Treg cells [10,11]. In patients with knee OA and accompanying obesity (BMI ≥ 30 kg/m^2^; ≥2 radiological grade according to Kellgren–Lawrence scale), metformin administration has been associated with slower cartilage volume loss in the medial compartment of the joint assessed via MRI after 4 years. These results suggest possible long-term beneficial effect in patients with OA and diabetes and potential for the disease-modifying action of metformin in OA that requires confirmation in future clinical trials [12].

The structural integrity of the tissues is maintained by fibroblasts, which produce extracellular matrix molecules [13]. These cells are morphologically almost identical to mesenchymal stem cells (MSCs). Their proliferation and differentiation capacities, as well as their surface phenotypes, are also very similar. It is likely that fibroblasts are mature cells derived from MSCs since the latter are considered to be more efficient [14]. In an experimental model of OA in rats, a positive therapeutic effect has been observed after the administration of MSCs cultivated with metformin. Metformin increases the migration potential of MSCs derived from adipose tissue, influences their immunoregulatory properties, and inhibits the production of proinflammatory and catabolic mediators involved in OA pathogenesis [15].

The knee is one of the most common localizations of osteoarthritic process. An association with metabolic syndrome and obesity is present in many cases, leading to the notion that the existence of different phenotypes of knee OA including metabolic knee OA, in which other complex mechanisms apart from mechanical joint overload, lead to disease progression. In this regard, there is increasing evidence for systemic effects of the adipokines that are molecules derived from the dysfunctional adipose tissue.

Adipokines are bioactive molecules such as leptin, resistin, adiponectin, and visfatin, acting as modulators of immune and inflammatory responses in OA both at local and systemic levels [16]. They are produced not only by cells of the adipose tissue, but also by immune cells, chondrocytes, and synoviocytes [17]. Leptin is involved in the regulation of adipose tissue volumes and body mass index (BMI), bone growth, immune responses, and proinflammatory mediators [18]. Increased leptin levels have been shown in many diseases, including in OA [19,20]. High levels of leptin are associated with the progression of disease in OA patients as they cause the activation of the matrix metalloproteases [19].

Serum, plasma, and synovial fluid resistin levels are also increased in OA patients compared with healthy subjects, suggesting a link between the inflammatory process and the altered metabolism of joint tissues [21]. Resistin levels in the plasma and synovial fluid of OA joints correlate with disease severity [22] and with the presence of inflammatory and catabolic factors in synovial fluid, such as matrix degrading enzymes, IL-6, and collagen type II C-telopeptide fragments [23]. Resistin is able to promote catabolic over anabolic activity in OA chondrocytes, as it modulates the expression of several microRNAs involved in the pathogenesis of OA [24]. Resistin promotes monocyte migration in the synovium [25] and upregulates the expression of pro-inflammatory mediators in chondrocytes in vitro [26]. Its serum levels correlate with the pain, progression, and severity of OA [27]. 

Collagenase-induced osteoarthritis (CIOA) is an experimental mouse model, induced by the intra-articular injection (i.a.) of collagenase, resembling some of the OA-like changes that occur in humans. It is a proper model with which to study fibrosis resulting from high numbers of fibroblasts, osteophyte formation, and the influx of inflammatory cells. According to previous studies comparing different mouse strains, ICR mice express most characteristics of human OA pathology [28]. In the present study, we aimed to investigate the effect of metformin, alendronate, and their combination on serum levels of the adipocytokines leptin and resistin, as well as on the expression of markers for fibroblasts (CD29), MSCs (CD105), and osteoclasts (RANK/RANKL) on bone marrow-derived cells in a mouse model of OA induced by the i.a. injection of collagenase. We hypothesized that the treated mice will display reduced clinical and histopathological signs of OA progression and thus, these drugs can be used for the treatment of OA patients.

## 2. Materials and Methods

### 2.1. Model of Collagenase-Induced Osteoarthritis (CIOA) and Treatment Regimen

Outbred ICR (CD-2) female mice, 8–10-weeks of age, with average body weights of 20–24 g, were used in the present experiments. All animal procedures were in accordance with the Guidelines of the Bulgarian Food Safety Agency (Protocol No 352/6 January 2012) and the international laws and policies (EEC Directive of 1986; 86/609/EEC, recommendation 2007/526/EC from European Community), and allowed by the Animal Ethical Committee at the Institute of Microbiology, Bulgaria. The animals were fed with standard pelleted chow and tap water ad libitum. OA was induced by i.a. injection with 2U/10 μL of collagenase from *Clostridium histolyticum* (Sigma-Aldrich, Saint Louis, MO, USA) under brief anesthesia (sodium pentobarbital 50 mg/kg, i.p.), assigned as day 0. The incidence of OA was approximately 80 to 90%. The sample size was determined by the formula *n* = log*β*/log*p*, where 1-β is the chosen power of 0.05 and *p* represents the proportion of the animals that did not develop CIOA (20%). Control groups of mice were i.a. injected with phosphate buffer saline (PBS) 10 times (every other day from day 0 to day 18). Four groups (10 mice per group) of mice were injected with collagenase. One of them was injected i.p. with PBS 10 times (every other day from day 0 to day 18). Another was injected i.p. with metformin (Sigma-Aldrich, Saint Louis, MO, USA) at a dose of 10 mg/kg or alendronate (Sigma-Aldrich, Saint Louis, MO, USA) at a dose of 20 mg/kg 10 times (every other day from day 0 to day 18). The last one was injected i.p. with 10 mg/kg metformin plus alendronate 10 mg/kg 10 times (every other day from day 0 to day 18). These doses were determined in preliminary experiments so no mortality was observed after 10 treatments (every other day from day 0 to day 18).

Bone marrow-derived cells were isolated by flushing femoral bones with complete RPMI-1640 medium (Sigma-Aldrich, Steinheim, Germany). The suspension was carefully aspirated to disrupt cell aggregates, followed by centrifugation for 5 min at 1000 rpm. Bone marrow precursors (1 × 10^6^/mL) were cultivated in 24-well plates in RPMI-1640 medium supplemented with 10% FBS, 2.5 mM HEPES, 5 mM glutamine, penicillin G (100 U/mL), and streptomycin (100 μg/mL) (Sigma-Aldrich, Steinheim, Germany) for 24 h. Adherent cells 1 × 10^6^/mL were divided into three groups as follows:

(1) cultivated in supplemented RPMI-1640 medium for 12 days and then subjected to FACS analysis for the determination of CD29+ and CD105+ populations;
(2) for osteoclast differentiation, adherent cells were cultivated in supplemented RPMI-1640 medium in the presence of 10^−7^ M vitamin D3 (1α, 25-Dihydroxychilecalciferol, Sigma-Aldrich, Darmstadt, Germany) for 6 days and RANKL expression was determined;
(3) for osteoblast differentiation, adherent cells were cultivated in supplemented α-minimal essential medium (α-MEM), containing L-ascorbic acid (50 μg/mL) and 5 mM β-glycerophosphate (Sigma-Aldrich, Darmstadt, Germany) for 14 days (mineralization medium) which was half changed every 3 days and then RANK+ cells were counted.

### 2.2. Flow Cytometry

At the end of cultivation, the cells (1 × 10^5^/sample) were washed with 2% FCS/PBS and incubated with antibodies against CD105 (clone MJ7/18, PE-labeled), CD29 (clone HMβ-1, FITC-labeled), RANKL (clone IK22/5, PE-conjugated), and RANK (CD265, clone 6D5, FITC-labeled). All antibodies were purchased from BioLegend (San Diego, CA, USA). Cells were acquired on a LSRII (BD Biosciences, Franklin Lakes, NJ, USA), and the data were analyzed using BD FACSDiva™ software (Becton Dickinson, San Jose, CA, USA).

### 2.3. ELISA Assays

Blood was collected by retro-orbital puncture and was allowed to clot for 1 h at room temperature. The concentration of resistin in sera was quantified by the Mouse Resistin (RETN) ELISA kit (Abbexa Ltd., Cambridge, UK) with a sensitivity of 46 pg/mL, and leptin was quantified by mouse ELISA kit (BioVendor, Brno, Czech Republic) with a sensitivity of 30 pg/mL.

### 2.4. Histopathological Analysis

At the end of the experiment, hind paws without skin were fixed for 3 days in 10% buffered paraformaldehyde (pH 7.0), decalcified by incubation in 10% ethylenediaminetetraacetic acid (EDTA) (in 0.1 M phosphate buffer, pH 7 for approximately 2 weeks, solution changed four times a week) [29], and embedded in paraffin. Sections (4 µm) were cut from the paraffin blocks by using a rotary microtome (Leica RM 2125 RTS, Leica Microsystems, Wetzlar, Germany) and stained with hematoxylin-eosin (H&E). All histological samples were prepared according to Schmitz et al. [30] and the microscopy analysis was performed using a light microscope connected to a digital microscope camera (Leica DM 2000 LED, Leica Microsystems, Wetzlar, Germany). Joint pathology was assessed using the OsteoArthritis Research Society International (OARSI) scoring system [31]. A six-degree (0–6) scale (OA grade) representing the severity or biological progression of the osteoarthritic process was applied. Moreover, a four-degree (0–4) stage scale (OA stage) was defined as the horizontal extent of cartilage involvement within one side of a joint compartment regardless of the main grade; Stage 1: <10% involvement; Stage 2: 10–25% involvement; Stage 3: 25–50% involvement; Stage 4: >50% involvement. Therefore, the score was calculated by OA grade and OA stage and thus represents a combined assessment of OA severity and extent. The observed joint changes for each specimen resulting in different scores were presented as average. Based on the score value, we proposed the following classification; early OA: OA score 0–12; established OA: OA score 12–24. Scoring was done by two independent researchers, blinded to the animal groups.

### 2.5. Statistical Analysis

Data were expressed as mean ± standard deviation (SD). Statistical analyses were performed by one-way ANOVA and the unpaired t-test, using InStat3.0 and GraphPad Software (La Jolla, CA, USA). Differences were considered significant when *p*  <  0.05.

## 3. Results

### 3.1. Inlfuence of Metformin and Alendronate on Bone Marrow Cell Differentiation

**Figure 1:**
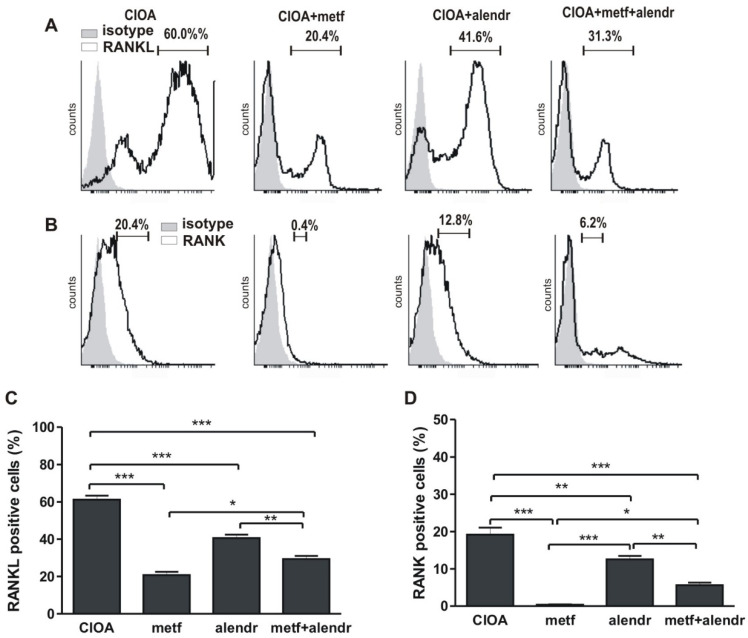
Flow cytometry data. (**A**) Percentage of RANKL+ cells. (**B**) Percentage of RANK+ cells. (**C**) Figure data of (**A**). (**D**) Figure data of (**B**). Data are means ± SD from three determinations (*n* = 10 per group). * *p* < 0.05; ** *p* < 0.01, *** *p* < 0.001, one-way ANOVA. (CIOA = collagenase-induced osteoarthritis; metf = metformin; alendr = alendronate).

Bone marrow (BM) cells were harvested from CIOA mice treated or untreated with substances at day 30 of arthritis. The stimulation with vitamin D3 resulted in a high percentage of RANKL+ cells (~60%) significantly suppressed by metformin (20.4%), alendronate (41.6%), or their combination (31.3%) (Figure 1A,C). The same tendency was observed in regard to RANK expression. A percentage of the BM cells from arthritic cells cultivated in mineralization medium were RANK+ (20.4%), decreased in the metformin-treated group (0.4%), alendronate-treated (12.8%), and combined group (6.2%) (Figure 1B,D). 

**Figure 2:**
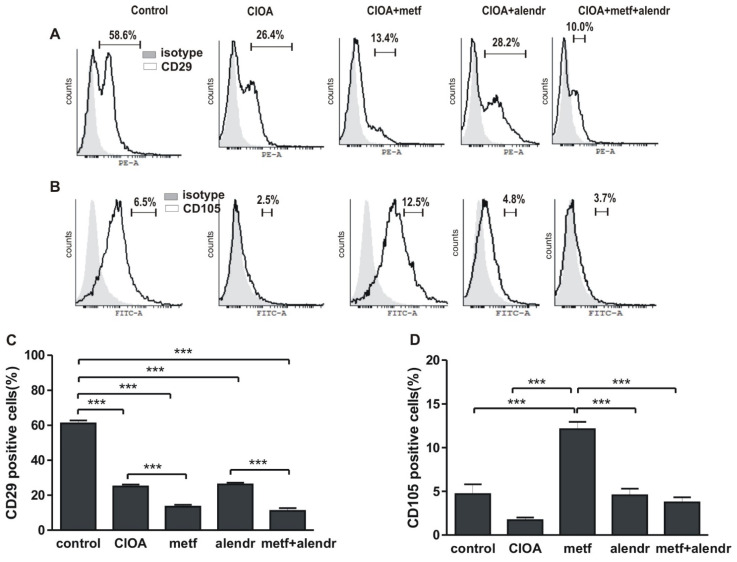
Flow cytometry data. (**A**) Percentage of CD29+ fibroblast-like cells. (**B**) Percentage of CD105+ mesenchymal cells. (**C**) Figure data of (**A**). (**D**) Figure data of (**B**). Data are means ± SD from three determinations (*n* = 10 per group). *** *p* < 0.001, one-way ANOVA. (CIOA = collagenase-induced osteoarthritis; metf = metformin; alendr = alendronate).

In the present investigation we defined as fibroblasts those expressing CD29 markers and as mesenchymal those expressing CD105 markers. We found that in the arthritic group the pool of fibroblast-like cells was decreased compared to non-arthritic (58.6% and 26.4%, respectively). Metformin and metformin+alendrondte enhanced this effect (13.4% and 10.0%, respectively), while alendronate had no effect compared to the CIOA group (28.2%) (Figure 2A,C). A relatively low number of CD105+ cells were observed in the control group (6.5%), with an even lower number observed for the CIOA group (2.5%). In metformin-treated mice the percentage of the CD105+ population was increased (12.5%) compared to all other groups (4.8% in the alendronate-treated group and 3.7% the in metformin+alendronate group) (Figure 2B,D).

### 3.2. Influence of Metformin and Alendronate on Serum Resistin and Leptin Levels

**Figure 3:**
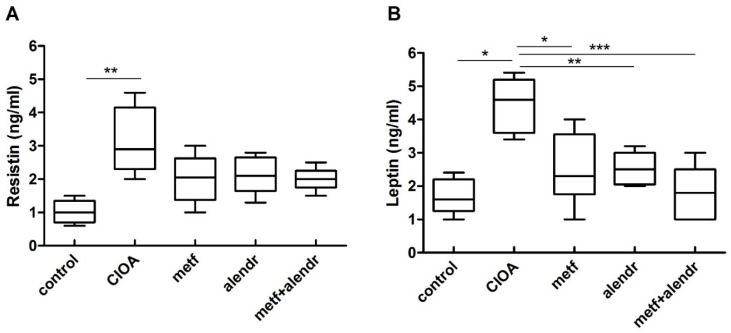
Serum resistin (**A**) and leptin (**B**) levels at day 30 of CIOA. Data are means ± SD from three determinations (*n* = 10 per group). * *p* < 0.05, ** *p* < 0.01, *** *p* < 0.001, one-way ANOVA. (CIOA = collagenase-induced osteoarthritis; metf = metformin; alendr = alendronate).

The data in Figure 3A show that in arthritic mice the serum resistin levels were elevated compared to control non-arthritic mice. No significant difference between the control and substance-treated groups was found. Although the resistin levels tended to be lower in the treated mice compared to arthritic mice, this difference was non-significant. Leptin serum levels were strongly increased in the arthritic animals compared to the control group. Metformin, alendronate, and metformin+alendronate treatment caused a significant suppression of leptin level (Figure 3B).

### 3.3. Histopathological Examination

**Figure 4:**
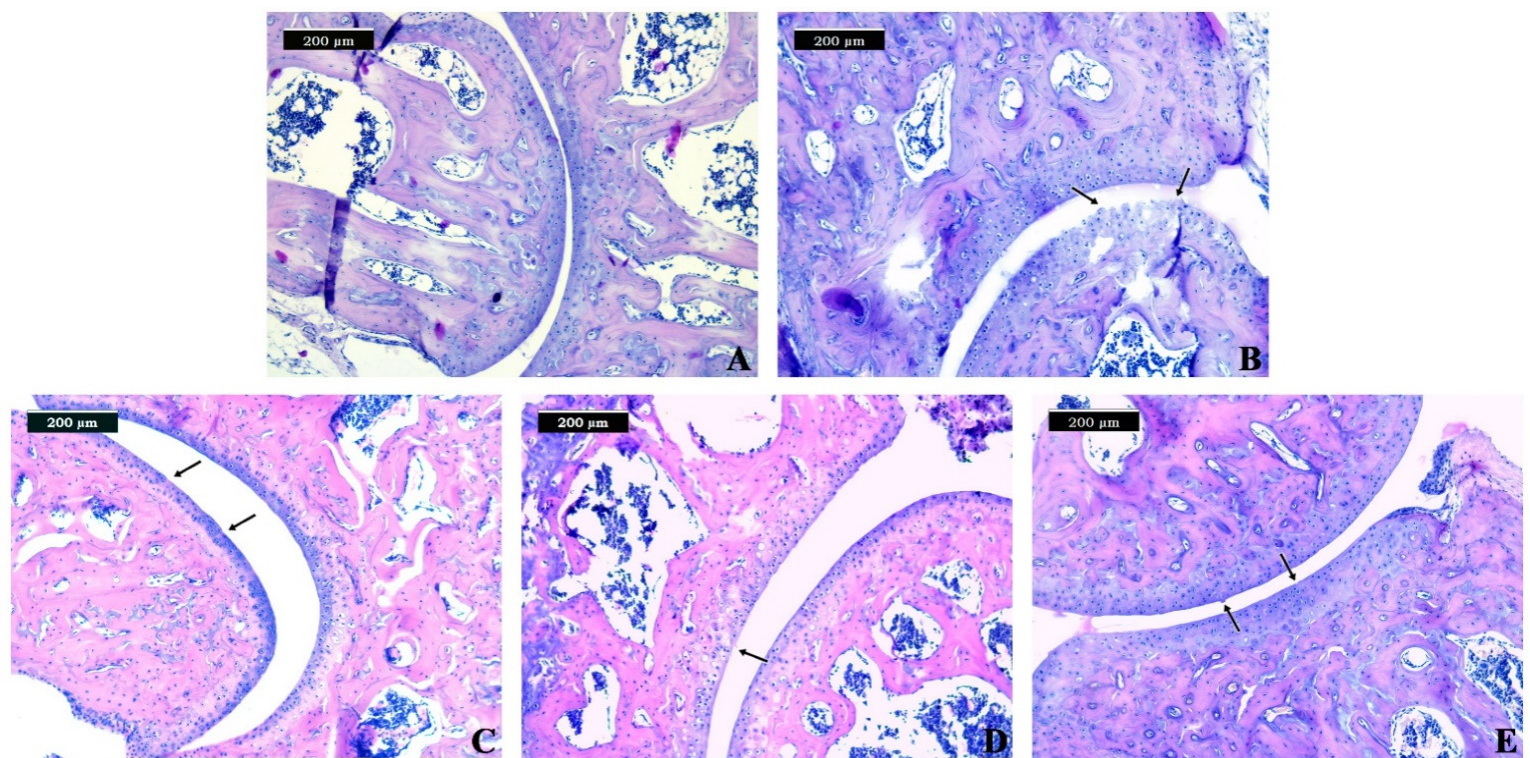
Effects of metformin and alendronate treatments on histopathological changes in the hind paws of collagenase-induced OA in mice. (**A**) Section of normal (control) hind paw showing normal architecture of the cartilage. (**B**) Section of untreated CIOA mice showing vertical fissures and matrix fibrillation extending into the mid zone. (**C**) Section of CIOA mice treated with metformin showing an uneven articular surface and small fibrillations. (**D**) Section of CIOA mice treated with alendronate showing focally fibrillation extending through the superficial zone. (**E**) Section of CIOA mice treated with metformin+alendronate showing an uneven articular surface and superficial fibrillation.

As stated by Glasson et al. [29], the histologic evaluation of OA in mice has increased exponentially in the past decade with the advent of transgenic animals being used to look for mechanisms involved in the development of OA. According to the authors, a universal system for the histologic scoring of murine OA would allow comparison of the severity of cartilage destruction across different spontaneous, enzymatic, chemical, or surgically-induced murine OA models. Our results from the conducted histopathological assessment are presented in Table 1 and Table 2, as well as in Figure 4. 

Generally, the observed changes in the joints were categorized in six main groups [31]: Grade 0: surface intact, cartilage morphology intact; Grade 1: surface intact; Grade 2: surface discontinuity; Grade 3: vertical fissures (clefts); Grade 4: erosion; Grade 5: denudation; and Grade 6: deformation. 

In the control group, we observed normal joint morphology (Table 1, Figure 4A). In this group, we found normal histological architecture of the joint, where the matrix and chondrocytes were organized into superficial, mid, and deep zones.

Regarding the regressive changes in the joint, in group 2, we observed Grade 3 changes (Table 1 and Table 2), which were associated with vertical fissures extending into the mid zone (Figure 4B). Necrosis was observed most prominently adjacent to fissures. The matrix texture is likely to become more heterogeneous, with adjacent domains of proteoglycan depletion and increased staining observed (Figure 4B). In groups 3 and 5, we established intact surfaces, resulting in small fibrillations without the loss of cartilage, which were categorized as Grade 1 (Table 1 and Table 2, Figure 4C,E). Moreover, we observed vertical clefts down to the layer immediately below the superficial layer and some loss of surface lamina in group 2 (Figure 4B). Within this grade, abrasion from shear forces leads to the loss of small portions of the superficial matrix parallel to the surface. Chondrocytes within the mid zone showed changes demonstrated by loss of orientation of the chondrons (Figure 4B).

OA stages were defined depending on the horizontal extent of the involved cartilage surface. In terms of the extent of the joint involvement (Table 2), we observed the highest extent in group 3, which showed more than 50% involvement. Unlike group 3, which had higher involved cartilage surface, the other groups showed milder tissue involvement as follows: groups 2 and 4 were categorized as Stage 3 (25–50% involvement); and group 5 was categorized as Stage 2 (10–25% involvement), respectively (Table 2).

According to the overall score, all groups were defined as demonstrating early OA (OA score range 0–12). The highest OA score and more severe alterations were observed in the joints of specimens from group 2 (Table 2). In group 4, the OA score was 6, determined by the proposed assessment scale. Moreover, the lowest OA score was observed in group 5, categorized as Grade 2.

## 4. Discussion

Although metformin and alendronate are known as potential anti-arthritic drugs, there are no investigations on their combined administration in clinical trials or experimental models. The chondroprotective effect of metformin is mediated by AMP-activated protein kinase (AMPK) signaling and the dysregulation of this pathway is related to many diseases including diabetes and osteoarthritis [12,32]. Additionally, metformin is an effective drug for suppressing OA pain [33]. However, Barnett et al. showed that treatment with metformin had no significant effect on OA progression [34]. Alendronate has been suggested as a prospective candidate for the treatment of OA patients, since platelet-rich plasma combined with alendronate delay OA progression by inhibiting the NF-*κ*B signaling pathway [35]. Evaluating the disease-modifying potential of metformin, alendronate, and their combination in an experimental model of OA, we will define the place of these drugs in the combined therapeutic strategy for OA patients. 

Most data regarding the impact of metformin and alendronate concern the later stages of OA development, potentially overlooking early OA when subchondral bone undergoes rapid resorption. In the present study, we demonstrated that metformin, alendronate, and their combination extenuate the active phase of OA. Bone remodeling includes osteoclasts responsible for bone resorption, and osteoblasts responsible for bone formation, strictly balanced in the absence of pathological processes. Osteoclasts are considered to be key players in arthritis, due to their ability to degrade the articular cartilage compartments of joints and osteochondral junction [36]. They are recruited in the subchondral bone during spontaneous OA progression by the classical RANK/RANKL pathway attracted by RANKL expressed by chondrocytes. These osteoclasts release cathepsin K collagenase that degrades cartilage and bone collagen [37]. Metformin is able to suppress osteoclastogenesis both in vivo and in vitro, decreasing the number of TRAP+ multinucleated osteoclasts. Its mechanism of action reveals the upregulation of RANKL expression in the inflamed joint and elevates osteoprotegerin and RANKL expression in osteoblasts [5]. Our results showed that treatment with metformin or alendronate, as well as with their combination, in the active phase of CIOA suppressed the expression of RANK on osteoblasts and RANKL on osteoclasts, as obtained by the ex vivo cultivation of bone marrow cells in mineralization or osteoclastogenic media. Therefore, these substances had a long-term effect on RANK/RANKL signaling and on the attenuation of arthritis. 

MSCs are reported to express markers such as CD73, CD90, and CD105, and are non-hematopoietic adherent cells that are able to differentiate into osteoblasts, osteocytes, chondrocytes, and adipocytes after stimulation with certain agents [5]. Fibroblasts are described to express the surface marker integrin beta-1 (CD29) and are spindle-shaped cells, producing an extracellular matrix that is responsible for maintaining the structural integrity of the tissue [4]. Alendronate expresses two effects of key importance for the late chronic stages of O, such as chondrocyte protection and inhibition of osteophyte formation [5]. In our study, early treatment with metformin or its combination with alendronate abated fibroblast differentiation in the chronic phase of CIOA, suggesting their relevance for the prevention of excessive extracellular matrix production and osteophyte formation. It is proposed that fibroblasts are aged MSCs [14]. Our results showed that metformin treatment was effective for the attenuation of fibroblast differentiation, but not of MSCs, while alendronate had an opposite effect. Nevertheless, the combination of metformin and alendronate had a suppressive effect on both MSC and fibroblast differentiation.

Increased serum and synovial levels of leptin have been reported in patients with OA and have been associated with disease severity and pain [38]. Leptin is involved in inflammatory and degenerative processes during OA, as it increases the production of proinflammatory cytokines and matrix-degrading enzymes [39]. Leptin expression is increased in the subchondral bone in OA, which is related to high levels of transforming growth factor β (TGF-β), alkaline phosphatase, collagen type I, and osteocalcin [40]. Treatment with metformin, alendronate, and their combination caused a significant decrease of leptin serum concentration in the chronic phase of arthritis, suggesting an advantageous effect via the restriction of both inflammation and remodeling processes in the affected joints. In terms of resistin, our results showed only a declining trend, but not significant differences between the treated and non-treated arthritic groups.

Histopathological scoring is used for the analysis of structural abnormalities and the severity of OA, reflecting the disorders of the cellular and molecular mechanisms of OA, as well as potentially identifying disease subgroups [41]. Histopathologically, alendronate treatment appeared to be less effective for CIOA restriction, which was in accordance with its weak impact on the count of fibroblasts and MSCs compared to metformin, or its combination with metformin. However, alendronate has been described to have a protective role for chondrocytes and inhibits osteophyte formation [5]. We suggest that the combination of alendronate and metformin can be used for inhibiting excessive bone resorption and aid in the preservation of bone structural integrity and function during OA.

## 5. Conclusions

The present study demonstrated that metformin, alendronate, and their combination attenuate the progression of osteoarthritis by inhibiting the expression of RANK and RANKL on osteoblasts and osteoclasts (after ex vivo culturing of bone marrow cells) and decreasing the serum concentration of leptin and resistin. In addition, taking in account the histological analyses, we found that the combination of alendronate and metformin may protect cartilage destruction that occurs during OA. Our data suggest that metformin combined with alendronate might be a useful approach for the treatment of OA patients.

## Figures and Tables

**Table 1:** Grade * of the histopathological alterations in the tested groups according to Pritzker et al. (2006).

Test Groups	Grade	Associated Criteria (Tissue Reaction)
**Group 1** (healthy control)	Grade 0 Surface intact, cartilage morphology intact	Normal architecture; the matrix and chondrocytes are organized into superficial, mid, and deep zones.
**Group 2** (CIOA)	Grade 3 Vertical fissures (clefts)	Vertical fissures extending into the mid zone, the matrix fibrillation extends vertically downward into the mid zone; cell death and proliferation may be observed most prominently adjacent to fissures.
**Group 3** (CIOA + metformin)	Grade 1 Surface intact	The articular surface is uneven and can demonstrate superficial fibrillation; this may be accompanied by cell death or proliferation; the mid zone and deep zone are unaffected.
**Group 4** (CIOA + alendronate)	Grade 2 Surface discontinuity	Focally fibrillation extends through the superficial zone to the superficial zone–mid zone portion; this may be accompanied by cell proliferation, decreased matrix staining, and cell death in the mid zone.
**Group 5** (CIOA + metformin + alendronate)	Grade 1 Surface intact	The articular surface is uneven and can demonstrate superficial fibrillation; this may be accompanied by cell death or proliferation; the mid zone and deep zone are unaffected.

* Grade—severity of the osteoarthritic process.

**Table 2:** Stage * of the histopathological alterations and OA score (OA score = grade × stage) according to Pritzker et al. (2006).

Test Groups	Stage	% Involvement (Surface, Area, Volume)	Grade	OA Score
**Group 1** (healthy control)	Stage 0	No OA activity seen	Grade 0	0
**Group 2** (CIOA)	Stage 3	25–50%	Grade 3	9
**Group 3** (CIOA + metformin)	Stage 4	>50%	Grade 1	4
**Group 4** (CIOA + alendronate)	Stage 3	25–50%	Grade 2	6
**Group 5** (CIOA + metformin + alendronate)	Stage 2	10–25%	Grade 1	2

* Stage—the horizontal extent of cartilage involvement within one side of a joint compartment regardless of the main grade.

## Data Availability

Data is contained within the article.
